# Physiological Basis of Genotypic Response to Management in Dryland Wheat

**DOI:** 10.3389/fpls.2019.01644

**Published:** 2020-01-10

**Authors:** Amanda de Oliveira Silva, Gustavo A. Slafer, Allan K. Fritz, Romulo P. Lollato

**Affiliations:** ^1^ Department of Agronomy, Kansas State University, Manhattan, KS, United States; ^2^ Department of Crop and Forest Sciences, University of Lleida - AGROTECNIO Center, Lleida, Spain; ^3^ ICREA (Catalonian Institution for Research and Advanced Studies), Barcelona, Spain

**Keywords:** wheat, *Triticum aestivum* L., nitrogen economy, yield components, crop management intensification, agronomic traits

## Abstract

A great majority of dryland wheat producers are reluctant to intensify management due to the assumption that lack of water availability is the most critical factor limiting yield and thus, the response to management intensification would be limited. We conducted on-farm field experiments across three locations and two growing seasons in Kansas using 21 modern winter wheat genotypes grown under either standard (SM) or intensified management (IM) systems. The goals of this study were to (i) determine whether the SM adopted is adequate to reach achievable yields by farmers in the region and (ii) identify differences in responsiveness to IM among a range of modern genotypes. Across all sites-years and genotypes, the IM increased yield by 0.9 Mg ha^-1^, outyielding the SM system even in the lowest yielding conditions. As expected, the yield response to IM increased with the achievable yield of the environment and genotype. Across all sources of variation, the yield responsiveness to IM was related to increased biomass rather than harvest index, strongly driven by improvements in grain number (and independent of changes in grain weight), and by improvements in N uptake which resulted from greater biomass and shoot N concentration. The IM system generally also increased grain N concentration and decreased the grain N dilution effect from increased yield. Genotypes varied in their response to IM, with major response patterns resulting from the combination of response magnitude (large vs. small) and consistency (variable vs. consistent). Genotypes with high mean response and high variability in the response to IM across years could offer greater opportunities for producers to maximize yield as those genotypes showed greater yield gain from IM when conditions favored their response. For the background conditions evaluated, intensifying management could improve wheat yield in between c. 0.2 and 1.5 Mg ha^-1^ depending on genotype.

## Introduction

Wheat (*Triticum aestivum* L.) is critical for food security as it provides c. 20% of calories and protein of human daily nutrition requirements (Shewry and Hey, 2015). It is the crop cultivated across the largest acreage in the world (more than 200 million hectares year^-1^; FAO-AMIS, 2018), and is mostly (80%) grown under rainfed conditions (FAO, 2003). Many of these regions produce rather variable, though overall relatively low, yields mainly due to the exposure to water stress. Rainfall in these regions is characteristically variable from season to season and is generally insufficient to maximize yield (FAO, 2003; Reynolds, 2010).

Farmers in most of these dryland regions are reluctant to intensify agronomic management. One major reason is the assumption that lack of water availability will limit yield potential and intensified management will provide no benefit, as expected from the Liebig's “law of the minimum.” However, this reluctance may be unjustified as several empiric and theoretical frameworks show the inadequacy of this “law” (De Wit, 1992; Sinclair and Park, 1993). In fact, crop yields could be enhanced when there is colimitation of different factors [i.e., when different resources are similarly limiting rather than when growth is severely limited by a single factor (Sadras, 2004; Cossani et al., 2010; Cossani and Sadras, 2018)]. The proven inadequacy of Liebig's “law of the minimum” implies that the most limiting factor could be used more efficiently when increasing the availability of other factors through intensifying management (Sadras, 2005). Moreover, the high costs of inputs and low wheat market prices drive farmers to reduce investments on crop management (Jaenisch et al., 2019). Thus, conservative behavior of farmers regarding intensification of management in dryland wheat regions may prevent them from achieving higher yields, even in the lowest yielding environments. Good empirical evidence of this is that Australian wheat yields have increased consistently due to reducing biotic stresses (nematodes) and increasing N fertilization (Passioura, 2002), even though water availability has not improved in Australia (Hochman et al., 2017).

Kansas is the largest winter wheat producing state in the US (c. 15% of the total US production, growing wheat in c. 3.4 Mha; USDA-NASS, 2018a), and experiences constraints to production which are typical of dryland wheat producing regions of the globe. Average farm yields have been relatively low (c. 3 Mg ha^-1^ during the past 30 years; FAO-AMIS, 2018) mainly due to highly variable, and overall scarce level of, rainfall (Lollato et al., 2017; Araya et al., 2019). Farmers in Kansas tend to be conservatively averse to risk, limiting the use of inputs due to the expectation on inconsistent yield responses. Perhaps contributing to this conservative behavior, wheat variety trials in Kansas evaluate the performance of genotypes under farmers' standard management rather than managing varieties for their yield potential. However, similar to other wheat regions (e.g., Cossani et al., 2011), there is empirical evidence in Kansas (Jaenisch et al., 2019) that wheat yields may improve by intensifying rainfed management practices.

The two major inputs that might be inadequately managed in standard management systems in Kansas are nitrogen (N) fertilization and chemical protection against foliar fungal diseases (Lollato et al., 2019a). Nitrogen fertilization rates in Kansas average c. 60 kgN ha^-1^ (USDA-NASS, 2018b), which is considerably lower than the estimated long-term agronomic optimum rate of the region (c. 90 kgN ha^-1^; Lollato et al., 2019b). Nitrogen limitation early in the growing season can reduce wheat tiller formation and survival, consequently reducing the number of spikes produced per unit area (Borghi, 1999; Montemurro et al., 2007) and the floret survival, resulting in reductions in grains per spike (Albrizio et al., 2010; Ferrante et al., 2013). Fertile tiller and grains per spike are major regulators of wheat yield (Slafer et al., 2014), thus lack of adequate N fertilization may limit water use and water use-efficiency (Asseng et al., 2001; Sadras and Roget, 2004; Cossani et al., 2012), even in dryland wheat production. Moreover, inadequate N availability during grain filling can reduce grain N concentration (Oury and Godin, 2007; Lollato et al., 2019a), which is a critical determinant of wheat end-use quality. Likewise, only about 25% of the wheat grown in Kansas is typically protected with foliar fungicides (USDA-NASS, 2018a). Severe incidence of foliar diseases can reduce wheat yield by lowering the source-sink ratio (Serrago et al., 2019). Moreover, even though the types and severity of fungal diseases (e.g., stripe rust [Puccinia striiformis f.sp. tritici] and leaf rust [Puccinia triticina]) vary depending on weather and genotypes, yield penalties due to diseases are common, as empirically evidenced by Jaenisch et al. (2019) and Lollato et al. (2019b). Furthermore, there has been an increase in stripe rust disease pressure and evolution of new pathogen races in recent years (DeWolf et al., 2017), which has challenged breeding programs to identify new sources of genetic resistance quickly. Thus, we believe that rainfed wheat in Kansas, and in dryland wheat growing regions in general, is likely grown under conditions that are chemically underprotected against foliar diseases that frequently reduce yield (USDA-NASS, 2018a) and where soil N availability is noticeably lower than the demand of the crop. Therefore, we hypothesize that current yields in this region are below those achievable under more intensive management in the form of higher N availability and chemical protection against diseases.

Although this hypothesis is proposed in general for modern wheat genotypes, different magnitudes of responsiveness to management intensification would be expected for specific genotypes. Thus, the hypothesis was tested considering a wide range of genotypes available to farmers in the region, allowing recognition of the level of genotypic variation and concurrently providing insight for breeding genotypes more responsive to intensive management. Future yield improvement in this (and any other) dryland region requires recognition of genetics characteristics underlying responsiveness to intensified management. Understanding agronomic traits associated with genotypic responses to management and yield determination can help breeding programs develop better adapted genotypes and enable producers to maximize yield while maintaining environmental quality.

We carried out field experiments with 21 modern winter wheat genotypes grown across three locations and two growing seasons in Kansas under either standard (SM) or intensified management (IM) systems to:

- determine whether the SM used in Kansas is adequate to reach achievable yields by farmers in the region by (i.a) quantifying the response to an IM system of improved N availability and protection against diseases, as well as, (i.b) ascertaining crop-physiological traits associated with yield responsiveness to IM across environments and genotypes; and
- recognize genotypic differences in responsiveness to IM among a range of modern cultivars, identifying degrees of overall responsiveness (expectedly from very responsive to mostly unresponsive) together with consistency in responsiveness to IM.

## Material and Methods

### General Experiment Information

Five rainfed field experiments were established in actual farmers' fields (i.e., the background conditions were those of real farms, not experimental fields) of three locations in Kansas (Conway Springs, Ellsworth, and McPherson) during two growing seasons: 2015–2016 and 2016–2017 (**Table 1**). The soil type was Bethany silt loam (fine, mixed, superactive, thermic pachic paleustoll) for Conway and Crete silt loam (fine, smectitic, mesic pachic udertic argiustolls) for Ellsworth and McPherson. The average yield recorded by farmers for the past 3–5 years before the establishment of the field trials in these fields was 3.3 Mg ha^-1^ for Ellsworth and 4.0 Mg ha^-1^ for Conway and McPherson.

**Table 1:** Experiment information. Site-years, plot coordinates, sowing and harvesting dates, previous crop, and total N rate (kg ha^-1^) for standard management (SM) at each location during the 2015–2016 and 2016–2017 growing seasons.

Year	Location	Coordinates	Sowing date	Harvesting date	Previous crop	N rate SM (kg ha^-1^)
2015–2016	Conway	37°27'34.94”N 97°37'43.33”W	10/13/2015	6/7/2016	soybean	157
	McPherson	38°15'56.99”N 97°35'34.04”W	10/7/2015	6/28/2016	wheat	106
	Ellsworth	38°35'37.99”N 98°19'58.18”W	10/7/2016	6/27/2017	wheat	67
2016–2017	Conway	37°27'36.7”N 97°37'48.3”W	10/11/2016	6/22/2017	corn	101
	McPherson	38°15'50.83”N 97°35'33.36”W	10/11/2016	6/20/2017	wheat	101

Conventional tillage was performed in the fall prior to wheat sowing in Ellsworth and McPherson, while a no-till system was used in Conway. Sowing and harvesting dates were within the optimal ranges in all cases (**Table 1**). Field trials were sown with a six-row Hege small plot cone drill. Plots were 4.6 m long and 1.5 m wide, comprised by six rows 0.25 m apart. At all sites, the seeding rate was 101 kg ha^-1^ [a weight-basis seeding rate being the usual recommendation for the region (Shroyer et al., 1997), due to the relative small variability in seed size among the most common cultivars]. Insect and weed occurrence was minimal and controlled with commercially available chemical products as needed. Weather data (**Table 2**) was collected daily (from sowing to harvest) from the Kansas Mesonet (http://mesonet.k-state.edu/) climate monitoring network from stations located near (c. 500 m) to the experimental sites. Soil fertility was evaluated within 2 weeks after sowing in all locations (**Table 3**). Soil samples were collected between plots to avoid plant and soil disturbance within plots, using hand-probes at 0–15 and 15–60 cm depth. At each depth, 15 soil cores were combined to represent the soil characteristics of each field experiment.

**Table 2:** Weather information. Cumulative precipitation (Cum PPT) in millimeters, maximum, minimum, and average daily temperature (T) in Celsius during the growing season and average of 30 years (1981–2011), cumulative growing degree-days (Cum GDD) in Celsius, and cumulative evapotranspiration (Cum ET) in millimeters per day at each location during the 2015–2016 and 2016–2017 growing seasons.

Year	Site	Season	Cum PPT (mm)	30-yr avg Cum PPT (mm)	T max °C	T min °C	T avg °C	30-yr avg T max °C	30-yr avg T min °C	Cum GDD (°C)	Cum ET (mm day^-1^)
2015–2016	Conway	Fall	189	148	15	2	*8*	14	1	699	148
		Winter	80	133	13	−2	*5*	10	−3	658	198
		Spring	494	324	26	13	*20*	25	13	1,919	447
	McPherson	Fall	117	125	15	2	*8*	14	1	963	159
		Winter	39	119	11	−2	*4*	12	−1	772	187
		Spring	351	325	25	12	*19*	24	11	1,982	426
2016–2017	Ellsworth	Fall	30	108	15	−1	*7*	13	−1	783	*NA*
		Winter	135	102	12	−2	*5*	9	−6	615	172
		Spring	239	276	25	11	*18*	24	11	1,573	383
	Conway	Fall	36	148	15	1	*8*	14	1	566	202
		Winter	187	133	13	0	*7*	10	−3	443	244
		Spring	332	324	25	12	*19*	25	13	1,284	391
	McPherson	Fall	43	125	14	1	*8*	14	1	524	151
		Winter	132	119	12	−1	*5*	12	−1	357	221
		Spring	217	325	24	11	*18*	24	11	1,170	405

There were no solar radiation data available for the fall period at the Ellsworth site, therefore cum ET in this location was calculated from January to June (harvesting). Fall; October to December, Winter; January to March, Spring; April to Harvest.

**Table 3:** Soil fertility information two weeks after sowing at each location during the 2015–2016 and 2016–2017 growing seasons.

	2015–2016				2016–2017					
	Conway		McPherson		Ellsworth		Conway		McPherson	
Depth (cm)	15cm	45cm	15cm	45cm	15cm	45cm	15cm	45cm	15cm	45cm
**pH**	6	5	6	6	6	7	6	6	6	6
**NO_3_-N (ppm)**	7	6	31	36	33	23	13	8	49	41
**NH_4_-N (ppm)**	13	6	27	13	16	15	8	6	16	14
**P_Mehlich (ppm)**	62	15	92	33	36	32	56	25	79	68
**K (ppm)**	239	231	383	243	365	301	226	251	370	309
**Ca (ppm)**	2,271	2,528	2,567	2,811	2,182	2,450	1,709	2,503	2,464	2,498
**SO_4_-S (ppm)**	19	15	14	10	16	7	6	6	11	10
**Cl (ppm)**	8	4	11	12	9	8	9	6	12	16
**CEC (meq 100g^-1^)**	22	25	19	21	21	16	22	24	22	20
**OM (%)**	3	2	3	2	3	3	3	2	3	3
**sand %**	25	21	15	12	18	13				
**silt %**	48	42	58	56	57	56				
**clay %**	27	37	27	32	25	31				

Soil test includes soil pH, nitrate- (NO_3_-N), and ammonium- (NH_4_-N) nitrogen, Mehlich-3 extractable phosphorus (P), potassium (K), calcium (Ca), sulfate-sulfur (SO_4_-S), chloride (Cl), cation exchange capacity (CEC), organic matter (OM), and percentage sand, silt, and clay in the soil at sampling depths from 0 to 15 cm and 15 to 45 cm.

### Treatments and Experimental Design

Twenty-one winter wheat genotypes, commercially available to farmers in the region (**Table 4**), were tested under two management practices at each location. The management systems tested were common farmer's practice (actual management made by the specific farmer in whose field the experiments were conducted) hereafter referred to SM versus IM. In the SM treatment, there was no fungicide application, and the N management (source, rate, and timing of application) varied slightly across fields depending on each farmer's practice (**Table 1**). In general, farmers applied N at planting and at early tillering stage (stage Z26 in the scale of Zadoks et al., 1974) in the spring with a total rate sufficient to achieve a yield goal of approximately 5 Mg ha^-1^, according to the recommendation guide from Kansas State University (Leikam et al., 2003). This rate considered soil N availability prior sowing in the topsoil layer (0–15 cm), soil NO_3_ in the profile (0–60 cm) (both shown in **Table 2**), previous crop credits, and tillage practice (Leikam et al., 2003). The IM treatment consisted of the SM treatment in each particular field with (i) an additional N rate of 45 kg ha^-1^ of N broadcasted as urea (46-0-0) at the onset of stem elongation stage (Z30), and (ii) two fungicide applications. The first fungicide application was made when the first node was detectable (Z31) to protect leaves and stems using a two mode of action product (24 g a.i. of fluxapyroxad ha^-1^ and 49 g a.i. of pyraclostrobin ha^-1^). The second fungicide was a three mode of action product (20 g a.i. of fluxapyroxad ha^-1^, 139 g a.i. of pyraclostrobin ha^-1^, and 82 g a.i. of propiconazole ha^-1^) applied at the heading stage (Z58) to protect upper leaves and spikes. The average yield produced under the IM treatment represents the water-limited achievable yield of site-years and genotypes, as defined by Evans and Fischer (1999).

**Table 4:** Information of agronomic traits [drought tolerance, maturity range (heading date), straw strength] and genetic resistance to most occurring fungal diseases in KS [leaf rust (*Puccinia triticina*), stem rust (*Puccinia gramini*), stripe rust (*Puccinia striiformis*), powdery mildew (*Blumeria graminis*), tan spot (*Pyrenophora tritici-repentis*), and Septoria tritici blotch (*Mycosphaerella graminicola*)] for the 21 genotypes tested in 2016 and 2017 growing seasons.

	Genotypes	Drought	Maturity	Straw strength	Leaf rust	Stem rust	Stripe rust	P. Mildew	Tan spot	Septoria
**1**	1863	6	5	7	7	1	3	6	6	6
**2**	AG Robust	*7*	3	2	4	5	2	*NA*	8	6
**3**	Bentley	*3*	4	6	5	2	5	6	6	5
**4**	DoublestopCL+	7	7	4	3	2	5	5	6	6
**5**	Everest	7	2	2	3	3	8	3	7	5
**6**	HotRod	7	3	2	3	5	4	4	6	5
**7**	Kanmark	5	5	1	2	3	6	7	6	6
**8**	Larry	6	6	3	7	2	2	5	5	6
**9**	LCS Chrome	5	8	3	2	2	3	6	4	4
**10**	LCS Mint	4	7	6	7	4	5	6	5	5
**11**	LCS Pistol	5	4	7	6	8	7	3	7	*NA*
**12**	SY Flint	5	4	4	6	3	4	7	7	7
**13**	SY Monument	6	7	5	2	2	2	5	5	4
**14**	T158	4	3	4	8	8	2	3	7	7
**15**	Tatanka	5	6	7	6	2	2	7	7	7
**16**	WB4303	*6*	4	1	3	1	4	5	6	6
**17**	WB4458	6	4	2	7	1	4	7	5	7
**18**	WBCedar	7	2	1	5	3	4	2	5	4
**19**	WB-Granfield	5	6	3	4	2	6	6	6	6
**20**	Winterhawk	4	6	3	7	8	6	5	6	7
**21**	Zenda	7	4	2	3	2	3	5	5	4

NA, not available due to insufficient information.

Legend for agronomic traits. Drought tolerance: 1 = excellent; 5 = good; 9 = poor. Maturity: 1 = early; 5 = medium; 9 = late. Straw strength: 1 = excellent; 5 = good; 9 = poor (high lodging risk). Legend for disease resistance levels: 1 = highly resistant, 3 = moderately resistance, 5 = intermediate, 7 = moderately susceptible, 9 = highly susceptible (DeWolf et al., 2017).

Treatments within each of the experiments were arranged in a split-plot design with genotypes assigned to the main plots and management to the subplots. Main plots were arranged in a randomized complete block design with three replications.

### Measurements

Aboveground biomass was sampled at physiological maturity from 0.5 m of a middle plot-row and the number of spikes counted before the material was fractioned into stover (leaves and stems), and spike (chaff and grains). Samples were dried at 60°C for one week, and then dry weights recorded. Spikes were counted and threshed; grains were weighed and counted to estimate yield and its numerical components: grain number per unit area and 1,000-grain weight on a dry weight basis. Samples were then ground (sieve 2 mm), and plant N concentration in stover and grains was determined *via* the LECO TruSpec CN combustion analyzer. The nutrient concentration of the chaff was estimated from that of the stover. Aboveground N uptake was estimated as the product between the weighted average of N concentration among organs by biomass and reported on a dry weight basis. Harvest index (HI) was determined as the ratio of grain yield by aboveground biomass at maturity. Nitrogen utilization efficiency was estimated as the ratio of grain yield by aboveground N uptake at maturity (Moll et al., 1982).

The severity of several foliar fungal diseases was evaluated in all experimental units approximately two weeks after each fungicide application. As the main goal of our study was to evaluate the management impacts on fungal diseases in general, our discussions will be based on the average incidence of all diseases found in each site-year.

### Statistical Analyses

Sources of variation in ANOVA comprised of genotype, management, site-year, and their interactions as fixed factors; and block nested within site and genotype nested within block as random effects, the latter to account for the split-plot design. Analysis of variance was conducted using the “lmerTest” package (Kuznetsova et al., 2017) in R software version 3.4.0. Descriptive statistics were calculated using the R package “doBy” (Højsgaard and Halekoh, 2016) and included mean, standard deviation (sd), and 0.25 and 0.75 percentiles for grain yield. To evaluate the impact of management on yield across genotypes and site-years, we built boxplots using the R package “ggplot2” (Wickham, 2009).

A biplot GGE model was used with yield, aboveground biomass, and HI as dependent variables to evaluate the genotypes performance and genotype and environment interactions across management and site-years (Romagosa et al., 2013).

We evaluated the relationships among measured variables by regression analyses using the “lm” function in the R package “ExpDes” (Ferreira et al., 2018). To estimate the impacts of agronomic traits on yield differences among environments and genotypes (i.e., the global responses), results are shown for all site-years and genotypes (n = 210), but also on average of genotypes for each site-year (n = 5), and on average of site-years across genotypes (n = 21).

Trait response to management within each particular background condition was estimated by subtracting the mean under IM by mean under SM. Likewise, the magnitude of genotypic yield responsiveness to management was evaluated as the difference between yield at IM and SM, averaged across background environments. The variability (i.e., lack of consistency) of genotypic response to management was assessed by the standard deviation of the mean yield response to management. The relationship between mean yield at IM and SM versus mean yield response to management was evaluated by regression analyses using the “lm” function in the R package “ExpDes” (Ferreira et al., 2018).

To investigate the causes of differences in N uptake due management we built a critical N dilution curve for each management system across all environments and genotypes by fitting the negative power function (Eq.1) suggested by (Justes et al., 1994).

(1) ShootNconcentration=a*biomass(−b)

where a is the shoot N concentration when biomass is equal to 1 Mg ha^-1^ and b is the dilution coefficient (i.e., rate of decrease in shoot N concentration as the biomass increases). We compared the intercepts and slopes of the relationship between grain N concentration and yield between IM and SM using the standardized major axis (SMA) analysis in the R package “smatr” (Warton et al., 2012).

### General Weather and Disease Incidence Conditions

For both 2016 and 2017 growing seasons, the average daily temperature was similar to the 30-year normal (1981–2000) of each region (NCDC-NOAA, 2019), except for winter season which was warmer than expected by approximately 3°C (**Table 1**). Precipitation during the fall of the 2015–2016 growing season was similar to the long-term in McPherson and slightly above average in Conway. Moderate drought and few freeze events were observed in the winter and early spring (around flag leaf emergence [mid-April]), which was then followed by greater than normal precipitation and below normal temperatures. During the 2016–17 growing season, fall months were drier and winter months were wetter than expected from an average year. The drier fall resulted in crops with less tiller formation (visually observed), which was then followed by a period of greater than average water availability and warm temperatures. In the spring (from flag leaf emergence and afterwards) weather was similar to those of an average year.

The fungal diseases recorded in the experiments at early season (stem elongation to flag leaf) were tan spot (*Pyrenophora tritici-repentis*), septoria tritici blotch (*Zymoseptoria tritici*), and powdery mildew (*Blumeria graminis* f. sp. Tritici). At late season, prevalent diseases were stripe (*Puccinia striiformis* Westend) and leaf rusts (*Puccinia triticina*). The greater leaf damage from foliar fungal diseases occurred after heading. Although average disease severity was similar across site-years (c. 10%), the overall disease pressure within an experiment varied due to differences in genetic resistance of genotypes. The disease severity recorded two weeks after heading under SM plots ranged from 4% to 38% in Conway 2016, from 4% to 20% in Conway 2017, from 4% to 50% in Ellsworth 2017, and from 2% to 27% in McPherson 2016. Under IM, disease severity ranged from 2% to 10% in Conway 2016, from 3% to 12% in Conway 2017, from 2% to 37% in Ellsworth 2017, and from 1% to 9% in McPherson 2016. No disease severity data was collected for McPherson 2017.

## Results

### Overall Effect of Management System on Crop Yield

**Figure 1:**
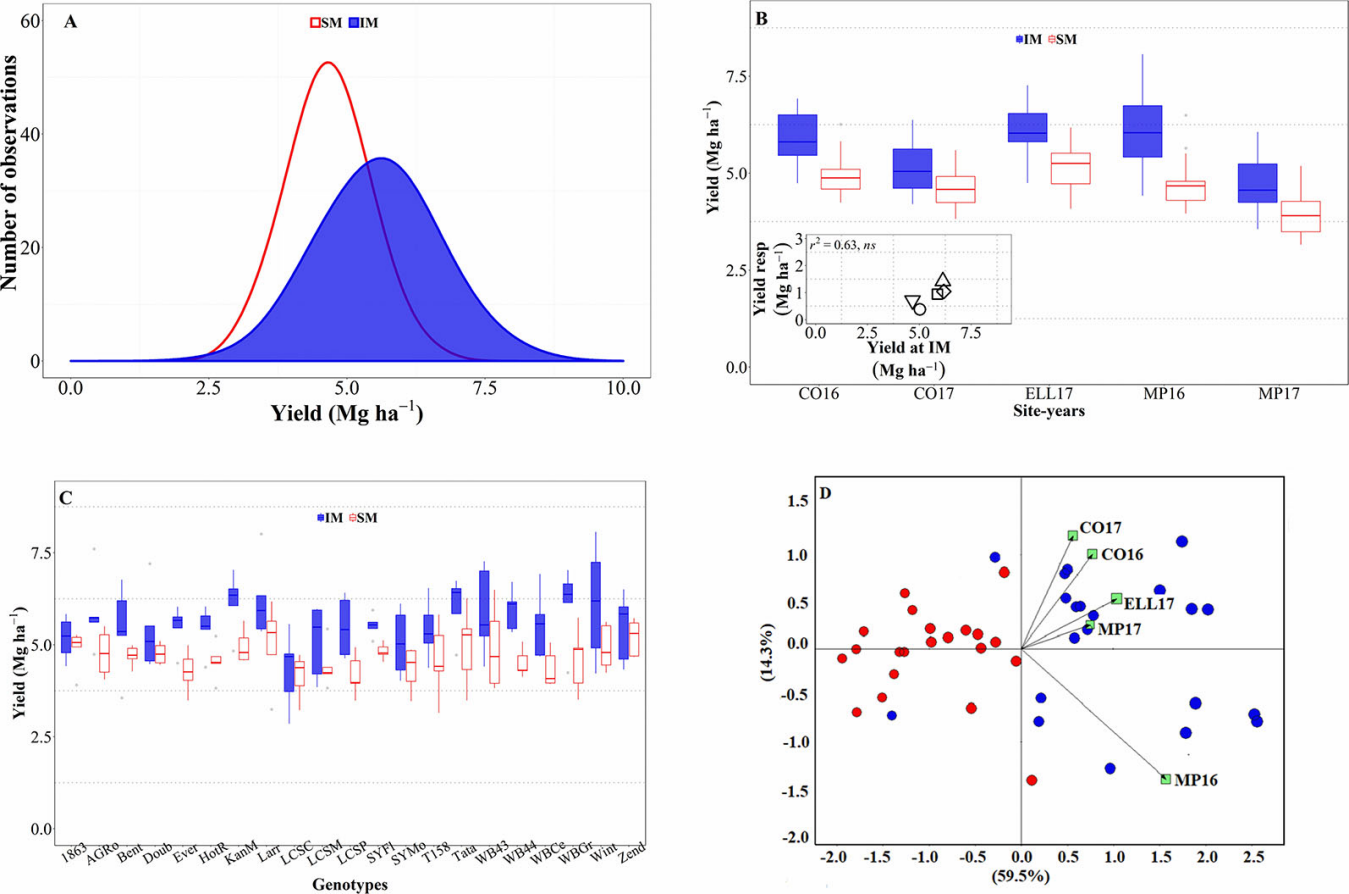
Distribution of the mean yield across environments and genotypes for intensive (IM) and standard (SM) management systems **(A)**. Mean yield for IM and SM systems on average of genotypes for each site-year **(B)**, with an inset showing the relationship between the average yield response to IM and achievable yield (yield in IM). Mean yield for IM and SM systems on average of site-years for each genotype **(C)**. Genotype and genotype × environment (GGE) biplot analysis for yield of 21 genotypes grown in five site-years at SM and IM systems **(D)**.

Across all sources of variation (five background environments given by the combination of sites and years and 21 cultivars grown in each of them), IM outyielded SM by an average of 0.9 Mg ha^-1^ (**Figure 1A**). Across the study, yields were normally distributed for both management systems and showed a larger standard deviation for the IM as compared to the SM (c. 0.97 and 0.67 Mg ha^-1^, respectively; **Figure 1A**). Usually, the lowest yields achieved in both systems tended to be similar while yields under IM were clearly larger than under SM in higher yielding conditions (**Figure 1A**). Therefore, the yield advantage of IM over SM was neither uniform across background environments (the interaction between management and site-year was significant at p < 0.05), nor across genotypes (although the interaction between genotype and management was significant only at a p = 0.14). The three-way (site-year x genotype x management) interaction was not significant (p = 0.81). However, the magnitude of the management effect was much larger than its interaction with the background environment (the mean square for management effect was more than tenfold higher than that of the site-year × management interaction), and therefore, that interaction was not crossover. That is, the IM always outyielded SM, though the magnitude of the difference was not uniform across sites-years (**Figure 1B**). Indeed, the response of wheat yield to the IM tended to increase with achievable yield (i.e., yield under IM) of the background environment (**Figure 1B**, inset). Regarding the overall differential response of the genotypes, we observed a consistent trend for IM outyielding SM in all genotypes, though that difference was not statistically significant in five out of the 21 genotypes (**Figure 1C**).

All these elements are clearly illustrated in the GGE biplot analysis (**Figure 1D**). In general, varieties under SM tended to have lower yields as compared to IM. The IM system seemed to have been better adapted, in terms of increased yield, than the SM across all site years (**Figure 1D**); although specific varieties were better adapted to certain particular background conditions.

### Traits Associated With Yield Responsiveness to IM

**Figure 2:**
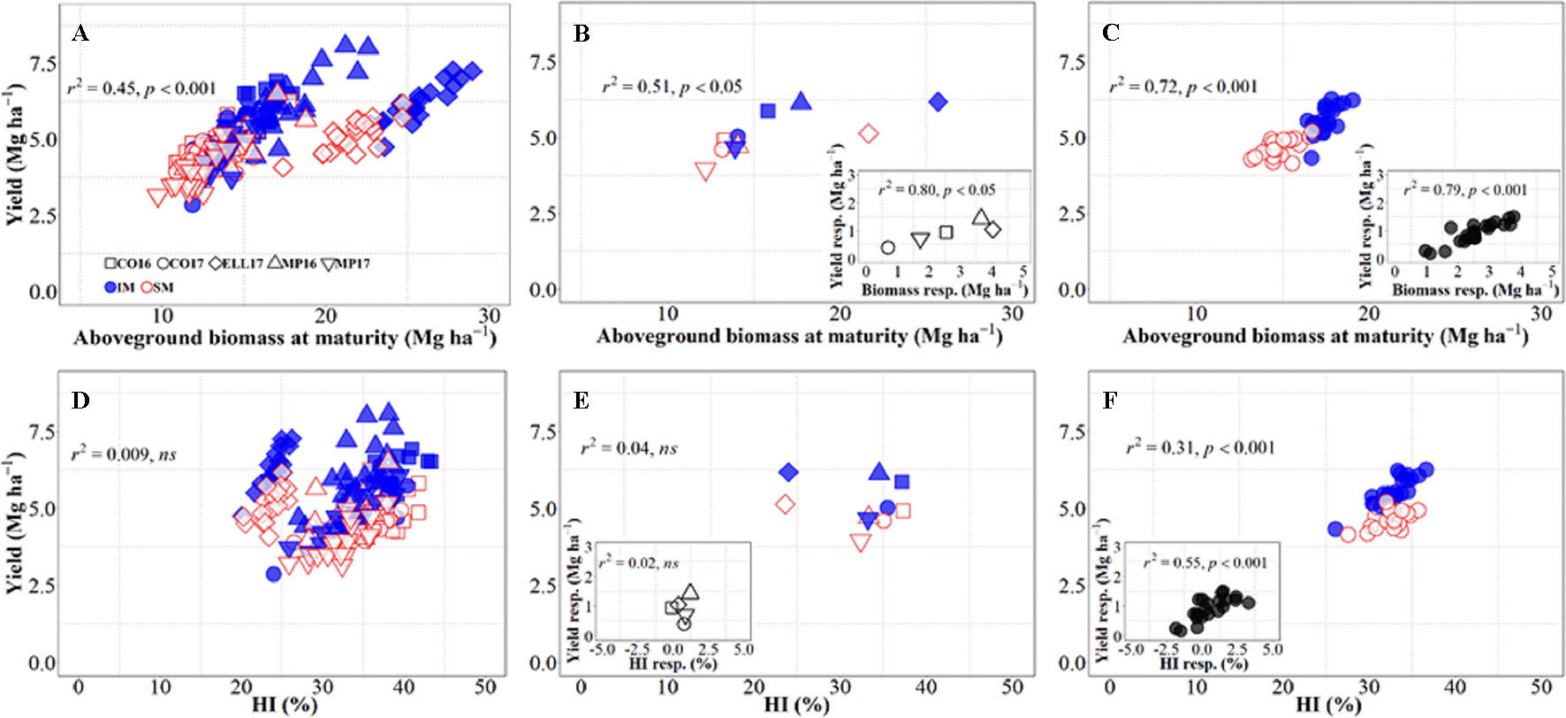
Relationship between yield versus aboveground biomass at maturity and harvest index across environments, genotypes, and management systems [intensive management (IM) and standard management (SM)] (n = 210) **(A, D)**, on average of genotypes for each site-year **(B, E)** (n = 10), and on average of site-years for each genotype **(C, F)** (n = 42). Insets are the relationships between the responses of the variables to intensive management (difference in the variable between IM and SM) averaged across either genotypes for each site-year (B, E insets) (n = 5) or site-years for each genotype (C, F insets) (n = 21).

There was an overall positive relationship between yield and aboveground biomass at maturity, with 45% of the variation in yield due to the combination of background environments, genotypes, and management systems explained by differences in biomass accumulation at maturity (**Figure 2A**), even though there was a clear penalty in harvest index in Ellsworth 2017 (Rhombs in **Figures 2A**, **B**, **D**, **E**). On the other hand, across all sources of variation considered, there was no relationship between yield and biomass partitioning toward the grains (**Figure 2D**), although this relationship was positive and significant within location-management combination (ranging from r2 = 0.14 in Conway 2016 to r2 = 0.60 in McPherson 2017) mainly driven by genotypic differences within each growing condition. Focusing on the background environmental conditions, the overall positive trend between yield and biomass demonstrates that differences in yield between site-years were in general due to differences in biomass accumulation (**Figure 2B**), and rather independent of site-year differences in harvest index across management systems (**Figure 2E**). Neither the relationship between yield and biomass, nor that between yield and harvest index, were significant within each management system (p > 0.05). It was clear, however, that biomass was more relevant than harvest index in explaining the differences in yield across sites-years, even within management systems (**Figures 2B**, **E**). Thus, the yield response to IM across sites-years was related differences between the two management systems for biomass rather than harvest index (**Figures 2B**, inset, **E**, inset). On the other hand, the yield differences between genotypes were significantly related to both biomass and harvest index across management systems, though the degree of association was substantially higher for biomass (cf. **Figures 2C**, **F**). Overall, it was clear that biomass responses to IM were the primary driver of the yield response of the genotypes. Evidence for this includes not only that coefficients of determination were more highly significant for biomass than for harvest index but also that while responses to IM of yield and biomass were always positive (**Figure 2C**, inset) in several cases, IM did not improve, and sometimes decreased, harvest index (**Figure 2F**, inset).

**Figure 3:**
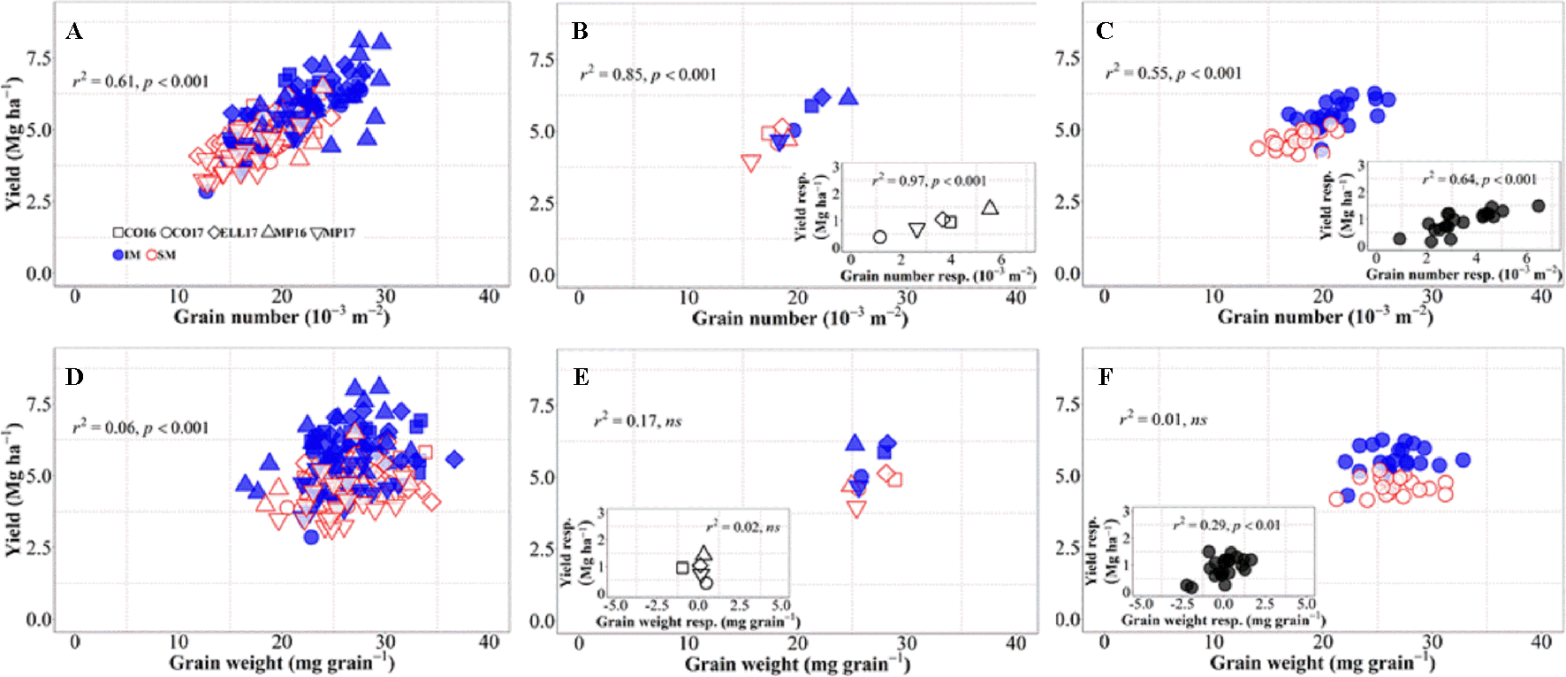
Relationship between yield versus grain number and grain weight at maturity across environments, genotypes, and management systems [ intensive management (IM) and standard management (SM)] (n = 210) **(A, D)**, on average of genotypes for each site-year **(B, E)** (n = 10), and on average of site-years for each genotype **(C, F)** (n = 42). Insets are the relationships between the responses of the variables to intensive management (difference in the variable between IM and SM) averaged across either genotypes for each site-year (B, E insets) (n = 5) or site-years for each genotype (C, F insets) (n = 21).

Changes in grain number per unit area explained 61% of the overall variation in grain yield, i.e., when accounting for environments, genotypes, and management systems together (**Figure 3A**). Although grain weight also significantly associated with differences in yield, the proportion explained was much lower (c. 6%, **Figure 3D**). Yield differences across environments were well explained by differences in grain number (**Figure 3B**), not only due to their high association across site-years (**Figure 3B**), but also because yield responses to IM within each of the site-years were strongly driven by improvements in grain number (**Figure 3B**, inset). On the other hand, differences in yield among environments were not explained by differences in grain weight within or across management systems (**Figure 3E**). Yield responses to IM of the different background environments were rather independent of those in grain weight (**Figure 3E**, inset). Indeed, there was almost no difference in grain weight between IM and SM within each of the site-years (**Figure 3E**), and therefore neither in the response of grain weight to IM (**Figure 3E**, inset). Similarly, differences in yield among genotypes across management systems were exclusively brought about by differences in grain number (**Figure 3C**), as the relationship with grain weight was negligible (**Figure 3F**). The relationship between yield and grain number across genotypes was strong within each of the management systems, but also the yield response to IM of the genotypes was associated with increases in grain number (**Figure 3C**, inset). The lack of relationship between yield and grain weight across genotypes and management was also true within each of the two management systems (**Figure 3F**). Even though the yield response of genotypes to IM was related to their grain weight response (**Figure 3F**, inset), the relationship could hardly be mechanistic as IM always improved yields even in situations where it decreased grain weight of several genotypes (**Figure 3F**, inset).

**Figure 4:**
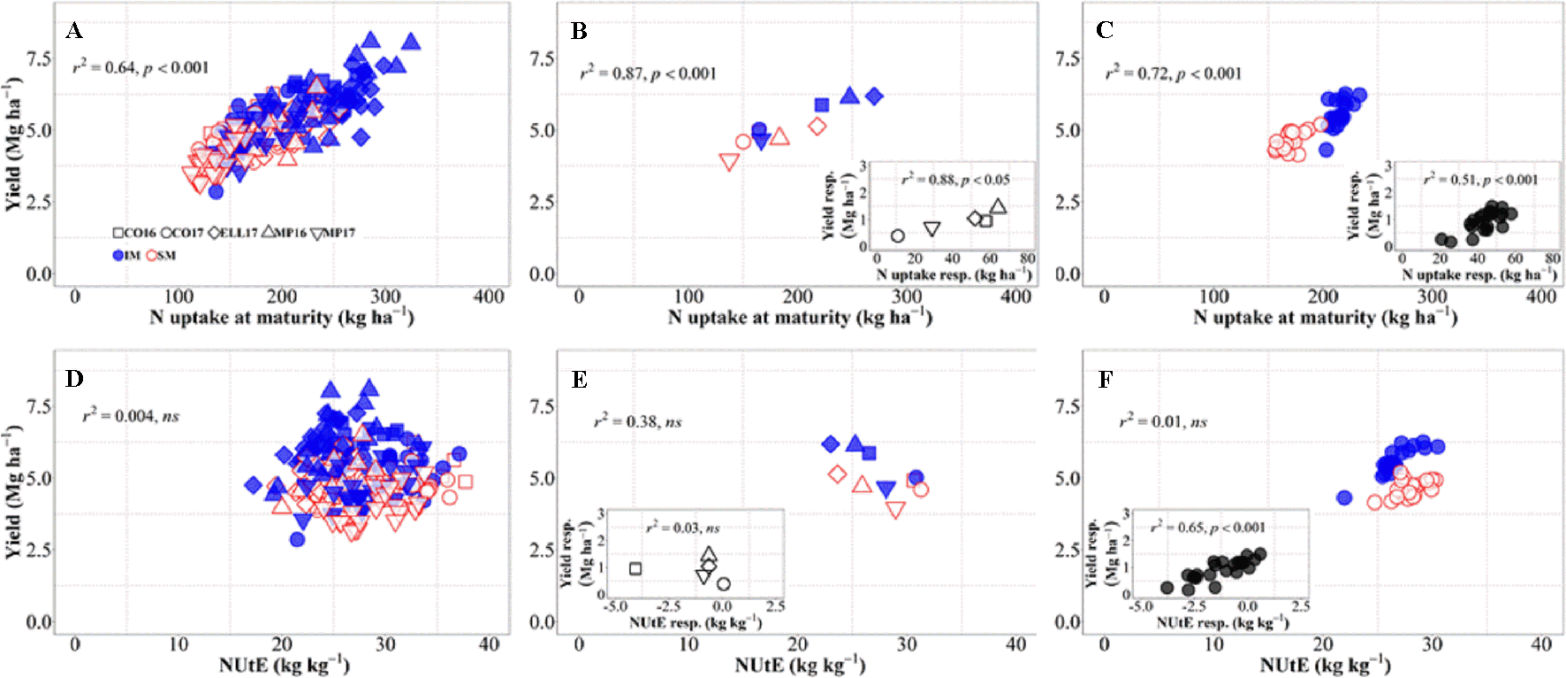
Relationship between yield versus nitrogen uptake and utilization efficiency at maturity across environments, genotypes, and management systems [intensive management (IM) and standard management (SM)] (n = 210) **(A, D)**, on average of genotypes for each site-year **(B, E)** (n = 10), and on average of site-years for each genotype **(C, F)** (n = 42). Insets are the relationships between the responses of the variables to intensive management (difference in the variable between IM and SM) averaged across either genotypes for each site-year (B, E insets) (n = 5) or site-years for each genotype (C, F insets) (n = 21).

There was an overall positive relationship between yield and N uptake at maturity. Differences in N uptake explained 64% of the variation in yield across background environments, genotypes, and management systems (**Figure 4A**). By dissecting the N uptake into shoot N concentration and biomass, we observed that differences in N uptake due to IM across site-years and genotypes were due to greater shoot N concentration under IM as compared to SM as biomass levels increased (**Supplementary Figure S1**). Conversely, changes in NUtE did not explain overall differences in yield across the entire dataset (**Figure 4D**). Considering only the differences between environments, there was a strong positive relationship reflecting that differences in yield among site-years were largely due to differences in N uptake across and within management systems (**Figure 4B**). Differences between sites-years in yield response to IM were related to their differences in N uptake response to IM (**Figure 4B**, inset). On the other hand, differences in yield between environments were not explained by their differences in NUtE (**Figure 4E**). In fact, there was a trend (p = 0.06) for site-years with higher yields to exhibit lower levels of NUtE (**Figure 4E**) and yield responses to IM of the different site-years was not mediated through NUtE response (**Figure 4E**, inset). Considering the differences between genotypes across management systems, there was also a positive relationship between yield and N uptake (**Figure 4C**), and differences among genotypes in yield response to IM were preceded by their differences in responses of N uptake (**Figure 4C**, inset). Yield differences between genotypes across management systems were not related to differences in NUtE (**Figure 4F**), but genotypic differences in yield within each management system were well explained by NUtE (**Figure 4F**) (p < 0.05, R2 = 0.78 for IM and R2 = 0.26 for SM). Although genotypic differences in yield response to IM were significantly related to their response in terms of both N uptake and NUtE, the former was the determinant of yield response, as NUtE was actually reduced (with most of values of NUtE response near or below zero) by intensifying management, partly compensating for the larger effect of management on N uptake relative to yield (**Figure 4F**, inset).

**Figure 5:**
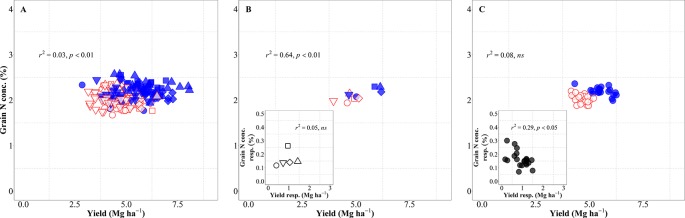
Relationship between grain nitrogen (N) concentration and yield across environments, genotypes and management systems [intensive management (IM) and standard management (SM)] (n = 210) **(A)**, on average of genotypes for each site-year **(B)** (n = 5), and on average of site-years for each genotype **(C)** (n = 42). Relationship between grain N concentration and yield responses to IM on average of genotypes for each site-year (**B** inset) (n = 5) and on average of site-years for each genotype (**C** inset) (n = 21).

The relationship between grain N concentration and yield was weak when considering all sources of variation together and IM improved both yield and grain N concentration as compared to SM, reducing the dilution of N in the grain (**Figure 5A**). This lack of relationship is actually hiding two contrary relationships, depending on whether the source of variation was site-years or genotypes. When considering the differences in site-years and management systems, the relationship was significantly positive, with changes in yield explaining 64% of the variation in grain N concentration across site-years and management systems (**Figure 5B**), mainly because IM improved both yield and grain N concentration in all five site-years (**Figure 5B**, inset). Conversely, changes in grain N concentration were not explained by differences in yield of genotypes considering both management systems together, though there was a significant negative relationship within management systems (**Figure 5C**) (p < 0.05; R2 = 0.33 for IM and R2 = 0.20 for SM). This implies that within management systems there was a general trend for higher-yielding cultivars to dilute the N in the grain and vice-versa. The fact that the relationship was not maintained when considering genotypes × management together reflects the positive effect of the IM system on both yield and grain N concentration. This may seem at odds with the fact that grain N concentration response to IM was negatively related to yield response of genotypes to management (**Figure 5C**, inset). However, the data were all in the positive quadrant: IM increased yields and grain N concentration of all genotypes; although there was a general trend for cultivars more responsive in yield to be less responsive in grain N concentration (**Figure 5C**, inset). Within each management system encompassing all sources of variation, the IM increased yield and maintained similar levels of grain N concentration while for SM there was a clear penalty in grain N concentration as yield increased (**Supplementary Figure S2** and **Table S1**).

**Figure 6:**
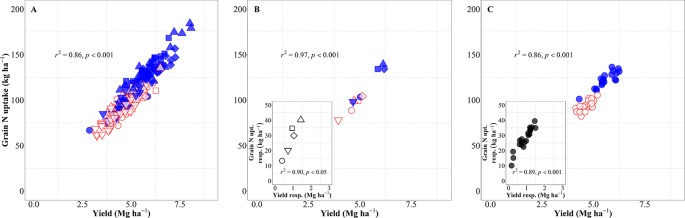
Relationship between grain nitrogen (N) uptake and yield across environments, genotypes, and management systems [intensive management (IM) and standard management (SM)] (n = 210) **(A)**, on average of genotypes for each site-year **(B)** (n = 10), and on average of site-years for each genotype **(C)** (n = 42). Relationship between grain N concentration and yield responses to IM on average of genotypes for each site-year (**B** inset) (n = 5) and on average of site-years for each genotype (**C** inset) (n = 21).

Yield (in terms of grain dry matter) was consequently a strong determinant of the total amount of N harvested (grain N uptake). Considering the overall variation due to background environments, genotypes and management systems, changes in yield explained 86% of the variation in grain N uptake (**Figure 6A**). This relationship was also very strong when focusing on either environment, both across and within management systems (**Figure 6B**), or genotypes (**Figure 6C**). The differences in grain N uptake response to IM, both between site-years (**Figure 6B**, inset) and between genotypes (**Figure 6C**, inset), mimicked the corresponding differences in yield responses.

### Genotypic Differences in Consistency of Yield Response

We restricted the analysis of the data so far to recognize differences and relationships across all sources of variation together or focusing on general responses to IM across sites-years (with averages across genotypes for each background condition) or across genotypes (with averages across background conditions for each genotype). This was done in order to determine whether an intensification of rainfed wheat management in Kansas would generally result in increased achievable yields and to assess the consistency of the outcomes (the first aim of the study). Nevertheless, genotypes varied specifically in their adaptation and responsiveness to IM. Examining overall responsiveness to IM was critical to draw general conclusions but also masked specific responses of particular genotypes. In this section we dissected these genotype- specific responses to IM, considering not only their responsiveness to IM but also their response consistency.

**Figure 7:**
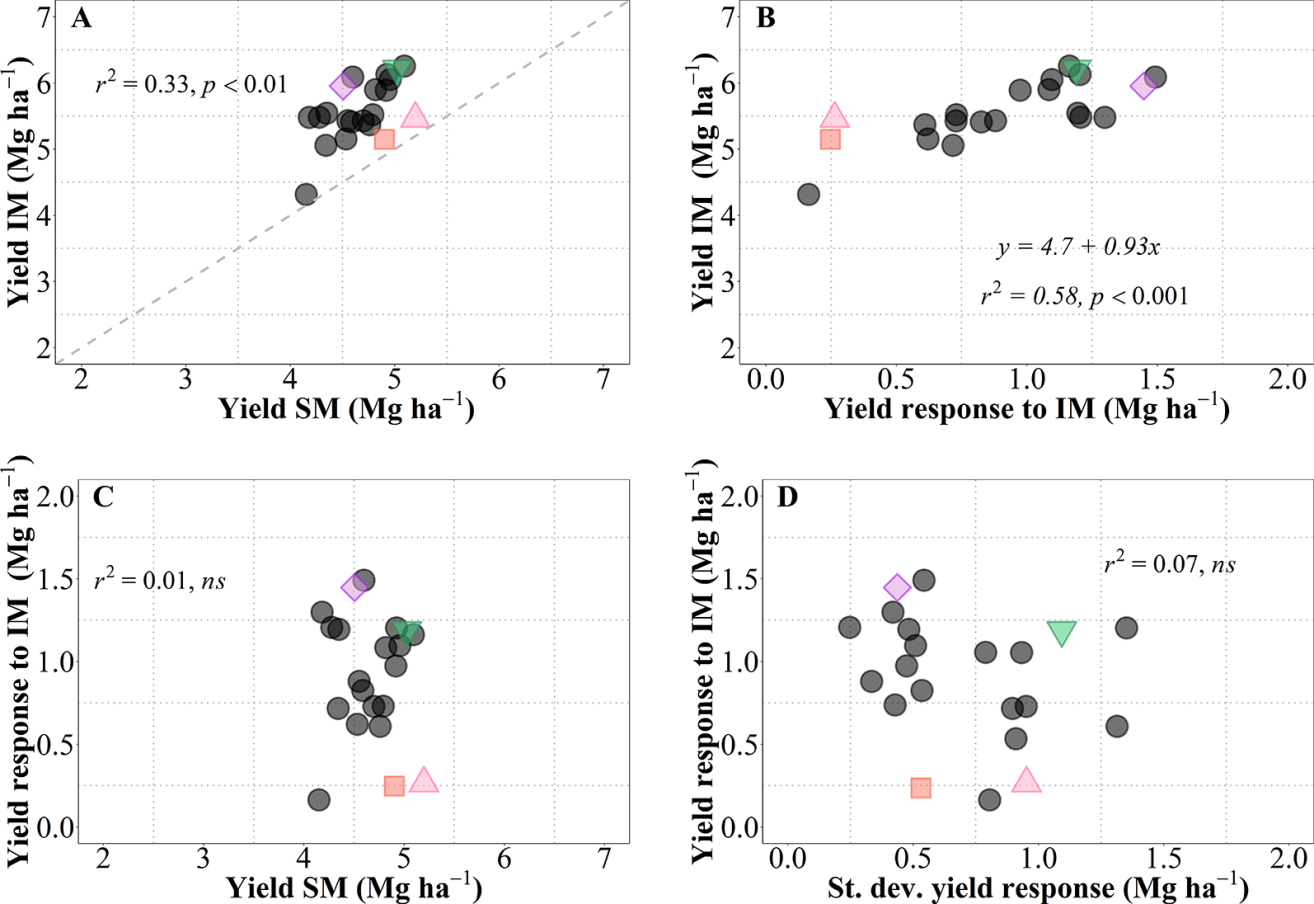
Relationship between mean yield under intensive (IM) versus standard management (SM) for the 21 genotypes tested averaged across site-years **(A)**. Relationship between mean yield under IM and yield response to IM (i.e., yield IM minus yield SM) **(B)**. Relationships between yield response to IM and either mean yield of SM **(C)**, or standard deviation of the yield response to IM **(D)**. The different symbols shows four genotypes selected to represent contrasting behaviors in terms of average responsiveness to intensive management (IM) and in stability of that responsiveness across all site-years selected genotypes, Zenda (triangle), Larry (inverted triangle), 1863 (square), and WB4458 (rhombus).

As mentioned above, we observed a generalized increase in yield due to IM in all genotypes, but with noticeable differences in magnitude and significance of the response (i.e., across all site-years yield increased between c. 0.2 and 1.5 Mg ha^-1^; this overall increase was statistically significant in 16 genotypes whilst only a trend in five genotypes; **Figure 1C**). This is reinforced by analyzing the yield of each of the 21 genotypes averaged across sites-years under both management systems (**Figure 7A**). As expected from overall results previously presented (**Figure 1C**), there was a considerable diversity in performance within each of the management systems, all data-points were above the 1:1 ratio (implying that all cultivars exhibited higher average yield under IM than under SM), and the performance of cultivars under IM depended largely on their responsiveness to intensification of the management (**Figure 7B**). It is relevant that performance of cultivars under IM was generally consistent with their performance under SM (in general, low- and high-yielding cultivars under IM were also low- and high-yielding cultivars under SM; **Figure 7A**). Even though the coefficient of determination was statistically highly significant, diversity in achievable yield and responsiveness to IM was still agronomically very significant, as evidenced by the 67% of the variation in IM not explained by that in SM. Thus, the overall response to IM across site-years included genotypes with relatively low responsiveness having either low (e.g., LCS Chrome), intermediate (e.g., 1863) or relatively high yield (e.g., Zenda) under SM; as well as genotypes with high responsiveness with either of the yield scenarios in SM (e.g., LCS Pistol, WB4458, Larry) (**Figure 7A**). Thus, the yield responsiveness to IM of the genotypes was largely unrelated to their performance under SM (**Figure 7C**), indicating that overall responsiveness to IM was mostly independent of adaptation to current management practices and thus achievable yield was strongly dependent upon the inherent genotypic responsiveness to IM (**Figure 7B**; please note that not only was the coefficient of determination highly significant but also that the slope was very close to one). Not only did genotypes vary in overall responsiveness to IM across site-years but also their differences in responsiveness were largely unrelated to their consistency in response to IM (inversely assessed by the standard deviation of their average response; **Figure 7D**). Although instability in response of the genotype did not contribute to the average yield in IM, it was naturally relevant to achieve the maximum yields that were equally related to the average response across sites-years and the instability in the response (**Supplementary Figure S3**). Being the variability in response (measured by the standard deviation of yield response to management) independent of the mean yield response (**Figure 7D**), maximum yields shall be obtained by genotypes combining a high average response and a high variability in response (**Figure S3**).

**Figure 8:**
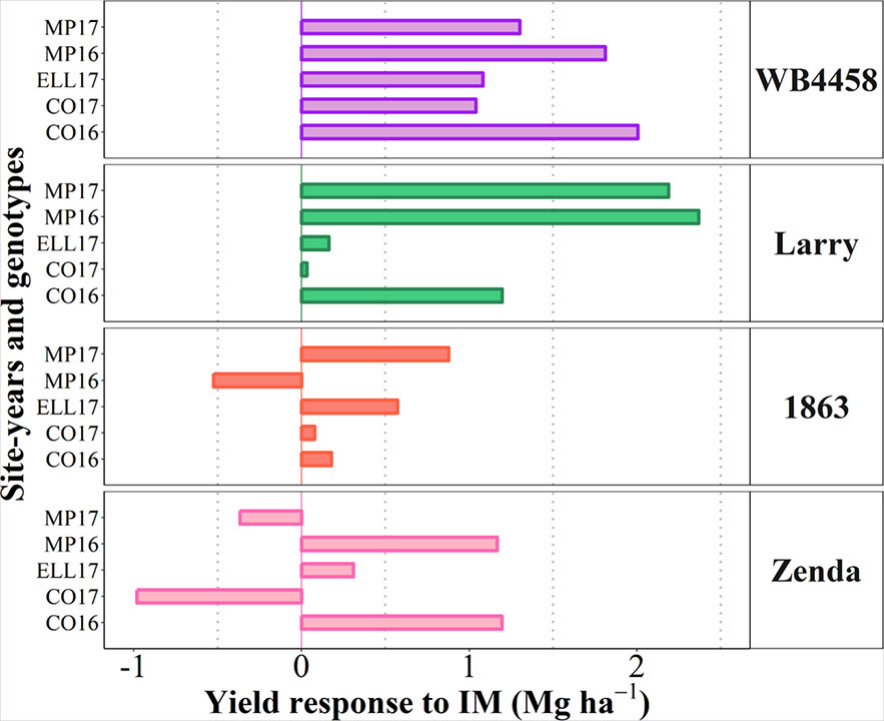
Yield response to intensive management (IM) for the selected contrasting genotypes at each individual site-year.

To illustrate the issue in more detail, we selected four cultivars representing contrasting average response to IM and contrasting stability in the response (**Figure 7D**). Cultivars 1863 and Zenda had both a small overall responsiveness but contrasted noticeably in consistency. Cultivar 1863 showed positive responses in four out of the five site-years, although with relatively small increases (from 0.18 to 0.87 Mg ha^-1^) and, in an exceptional case, showed a yield penalty though the magnitude was small (c. 0.52 Mg ha^-1^; **Figure 8**). On the other hand, due to its instability in response Zenda had c. 1 Mg ha^-1^ decrease in yield in Conway 2017 but also more than 1 Mg ha^-1^ yield gain in both Conway 2016 and McPherson 2016, and marginal responses in the other two environments; **Figure 8**). The same sort of lack of uniformity in consistency across sites-years was evident for genotypes with larger average responsiveness. For instance, cultivars such as WB4458 had simultaneously high and stable responsiveness to IM (**Figure 7D**), therefore responding with noticeable improvements in yield across all five site-years (ranging in response from 1 to 2 Mg ha^-1^; **Figure 8**). Meanwhile, genotypes such as Larry were highly responsive to management on average, but their response was not stable across site-years, with a very large response in some environments (> 2 Mg ha^-1^ yield gain in McPherson 2016 and 17), a high response in other environments (> 1 Mg ha^-1^ gain in Conway 2016), but mostly unresponsive in the other two site-years (**Figure 8**).

## Discussion

Results reported in this paper come from a study carried out in real farmers' fields. Working in realistic farming systems instead of carrying out experiments in experimental stations implies accepting restrictions in experimental procedures and produce “noisier” datasets, such as slightly different background environments for the standard treatment; but has a clear advantage when conclusions are expected to be pertinent (Rzewnicki et al., 1988). Moreover, conclusions were reached based on a very simplistic approach of applying a single intensification measure against what farmers were actually doing regardless of the particular situation. The aim was to test yield responses to management across different site-years to determine whether farmers are too conservative and thus missing opportunities of achieving greater yields. Naturally, an optimal level of intensification would likely be different for particular fields. Therefore, this paper does not contribute a tool to define the level of intensification required but only to uncover whether or not the current level of intensification is too conservative, evidencing whether or not there are opportunities to increase yield from the baseline. Similar to our data, several studies have registered average achievable yield for the region of c. 5.5 Mg ha^-1^ in field experiments (Lollato and Edwards, 2015; Jaenisch et al., 2019), simulation studies (Lollato et al., 2017), and survey of yield contest fields (Lollato et al., 2019b).

### Intensifying Management to Increase Rainfed Wheat Yield

Intensification of management practices and adoption of genotypes highly responsive to management can contribute to increasing wheat yields required for achieving food security, while improving the relatively low N use efficiency of production systems (Raun and Johnson, 1999). However, following a more conservative approach, dryland-wheat producers have been traditionally reluctant to intensify crop management and frequently prefer growing “stable” genotypes that are expected to perform relatively well under conservative conditions but are less responsive when under better growing conditions (i.e., intensified management, and fertile soils).

Climate variability affects the performance of genotypes and their response to management, challenging an effective implementation of management practices across seasons. Changes in precipitation (e.g., amount, intensity, and timing) and temperature patterns may interfere with crop adaptation (Reynolds and Ortiz, 2010), availability of resources (Chloupek et al., 2004), and enable conditions for pests to develop (Agrios, 2005; Legrève and Duveiller, 2010). Although the factors above may explain the variation in yield response to management across site-years, there was no single background condition in our study in which wheat yield, averaged across the 21 cultivars considered, decreased in response to IM. This suggests that, for the background environments evaluated, an excessively conservative attitude regarding the intensification of agronomic management is restricting farmers-yield in the region. Similar results were shown for rainfed wheat in other dryland regions (McDonald, 1989; Connor et al., 2011) as well as in other studies in the same region (Dorsey, 2014; Jaenisch et al., 2019; Lollato et al., 2019b). While we characterized the physiological basis of yield response to IM, future studies could focus on yield comparisons between IM and SM on a large number of fields to determine the most often probability of yield response and perhaps the magnitude of the yield gap.

Adequate N availability during the growing season is critical for wheat grain yield and quality (Entz and Fowler, 1989). There is usually a curvilinear relationship between yield and N rate (Simpson et al., 2016), but this relationship depends on yield potential (Savin et al., 2019) and might be linear or nonexistent (Lollato et al., 2019b). In the present study, yield was improved due to N rate and positively associated with higher N uptake and grain number, similar to previous reports which also suggested an increase in water use-efficiency (Entz and Fowler, 1989). Determining the agronomic optimum N rate is challenging in rainfed cereal production due to the variability in growing season precipitation and yield potential (Lollato et al., 2017), and leads to a dominant producer-mindset based on Liebig's “law of the minimum” that induces to underfertilize (Connor et al., 2011). Thus assuming (correctly) that water is commonly the most stressful factor limiting yield, it is overlooked that N availability may well improve water use and water use efficiency (Sadras, 2004; Cossani et al., 2012). The other factor supporting reluctance to fertilize rainfed wheat is the idea that it may bring about “haying-off” (i.e., an expected negative yield response to N fertilization of dryland wheat; van Herwaarden et al., 1998). However, it seems that this effect has been consistently reported only in Eastern Australia; as in other dryland regions this yield penalty is not evidenced beyond exceptional cases, and yield gains are frequently reported (Palta and Fillery, 1995; Asseng and van Herwaarden, 2003; Cossani et al., 2011) in line with results reported herein, with the exception of the cultivars with low overall responsiveness that may eventually exhibit a yield penalty (once again the “conservative” attitude of selecting “stable” cultivars induced to the very few cases of “haying-off” reported in the present study.

Moreover, the appearance of new populations of fungal diseases able to break genetic resistance of modern wheat genotypes (Chen, 2005) can result in need of fungicide application, in some cases even for relatively new cultivars that are expected to be resistant. The magnitude of yield loss from lack of fungicide varies according to the disease pressure, weather, fungicide management (i.e., timing and source), and genetic resistance (Thompson et al., 2014; Lopez et al., 2015; Benin et al., 2017). Naturally, years with considerable disease pressure will result in greater yield response to fungicide (Cruppe et al., 2017; Jaenisch et al., 2019) on cultivars susceptible to the most prevalent disease in the season (Thompson et al., 2014). However, we showed that yield advantages of a management intensification, including fungicide protection, produced yield gains across a range of sites-years and modern cultivars. This indicates that in most conditions of this dryland region, the penalty imposed by foliar diseases would be significant and fungicide application would be economically viable to producers (at least within the site-years evaluated in this study and other years with similar growing conditions). Furthermore, we found a positive relationship between the yield response to IM and the achievable yield under IM, which agrees with literature suggesting that the magnitude of responses to N and fungicide applications depend on the environmental yield potential of the growing season (Cruppe et al., 2017; Lollato et al., 2019b). Thus, it seems that the consequences of the aversion to risk are worse in conditions of higher achievable yield, which can be detrimental for further yield progress.

### Relevance of Yield Determining Traits in the Response of Wheat to Intensive Management

The magnitude and consistency of yield response to agronomic management can vary due to physiological aspects (e.g., ability to produce greater yields per unit of N supplied [NUE]) (Russell et al., 2017) and adaptation patterns of genotypes across different environmental conditions (Chloupek et al., 2004; Barraclough et al., 2010). In line with our results, other studies have found that genotypes more responsive to N management have greater biomass accumulation and N uptake at maturity (Kanampiu et al., 1997), and that their differences in yield are associated with differences in HI through differences in grain number produced per unit area (Calderini et al., 1995). The response of genotypes to N can be associated with their high yield potential and N use efficiencies (Ortiz-Monasterio et al., 1997). Grain yield improvements due to N management was achieved by increasing N uptake at maturity (López-Bellido et al., 2005), through improving N uptake efficiency (Barraclough et al., 2010) or utilization efficiency of genotypes (Cossani et al., 2012). However, reduction in NUtE is expected when improvements in N uptake from management occur at larger magnitude relative to yield (Gaju et al., 2011). In our data, yield increases due to IM occurred through improvements in N uptake, and the greater increase in N uptake from IM relative to yield reflected a reduction in NUtE. Although IM improved both yield and grain N concentration, genotypes with large yield gain from IM showed a reduction in grain N concentration (Giunta et al., 2019; Lollato et al., 2019a). Overall, our experiments were conducted during two growing seasons resulting in overall low grain protein concentration under SM and improved grain protein under IM, suggesting an opportunity to increase yield and maintain quality with IM. Previous research has proposed a critical value for grain protein concentration of 11.5% above which yield is not limited by N for hard red winter wheat in the region (Goos et al., 1982). In our study, average grain protein concentration for SM and IM were 11.5% and 12.5%, respectively. Thus, considering the narrower range of yield values (from 0.7 to 4 Mg ha^-1^) in the latter study as compared to our data (from 3 to 8 Mg ha^-1^) and the N dilution process in larger grain dry matter (Justes et al., 1994), we could postulate that yield was somewhat limited by N under SM in our study, and additional; N application would increase farmer's net return. A broader range of N rates would have to be tested to definitively make such conclusions.

Top-dress N application at late tillering stages improves yield by increasing grain number per unit area (Ercoli et al., 2013). Therefore, yield differences among genotypes are usually explained by differences in grain number as compared to grain weight at maturity (Arduini et al., 2006). The larger plasticity of grain number relative to grain weight (Sadras and Slafer, 2012; Wang et al., 2017) likely plays a role in this observation and may clarify our findings where grain number was the main yield component contributing to the response of genotypes to management (Slafer et al., 2014). Furthermore, the possible increase in late-season tiller production and survival from the N and fungicide applications may have resulted in additional formation of smaller spikes with smaller grains. Thus, the overall decrease in grain weight due to IM could be attributed to the larger number of smaller grains resulting from the late tillers, consequently decreasing the overall average grain weight in the IM relative to the SM (see Acreche and Slafer, 2006).

In general, the impacts of management on the performance of genotypes are evaluated for a small set of genotypes (Russell et al., 2017), and information about the scope of physiological determinants of genotypic responsiveness to management is usually limited. Our study utilized a large set of modern wheat genotypes differing in agronomic traits and genetic origin and characteristics, and thus, it provides insights on physiological mechanisms associated with response to the management of modern winter wheat genotypes.

Producers could consider approaches regarding the risks of intensifying management. The more risky approach is to grow genotypes with high average responsiveness to management and high variability on the response (i.e., unstable, as the standard deviation of the response was positively related to yield under IM, **Figure S3**) while the less risky approach is to grow genotypes with high mean response but stable yields in response to management. The former indicates that farmers who are willing to accept some risks to maximize productivity should select genotypes with unstable response, as those are the ones that maximize yield when the conditions favor response. In general, high yielding genotypes tended to be more unstable although with greater chance to maximize yield than low-yielding genotypes (the concept of stability can be also seen as lack of responsiveness to improvements in growing conditions; Calderini and Slafer, 1999). This is similar to the findings of Grogan et al. (2016) in which phenotypic plasticity (or the opposite of stability) of grain yield was a positive trait for 299 hard red winter wheat genotypes evaluated in the Great Plains. Indeed, breeding programs tend to select under more favorable conditions than those representing the average of the target population of environments in which the released cultivars are to be grown (**Box 1**). This is because cultivars of higher yield potential tend to outyield low-yield potential cultivars under a rather wide range of conditions (Slafer et al., 2005 and references quoted therein). Accordingly, Voss-Fels et al. (2019) demonstrated that breeding for genotypes under high input levels resulted in genotypes able to outperform under both high and low input levels. Understanding the physiological bases at the crop level of organization determining yield can help guide breeding to select prospective parents to produce strategic crosses aiming to increase the genetic gains in yield (**Box 2**), which would in turn require higher levels of intensification of management to reach the achievable yield of the newer cultivars produced. Thus, through understanding performance and responsiveness capacity of new genotypes, breeding programs would be more likely to identify genotypes with relatively good yield under standard conditions, but highly responsive when resources are available.

Box 1 **Relevance of High-Yielding Selection Environment.**

**Figure B1:**
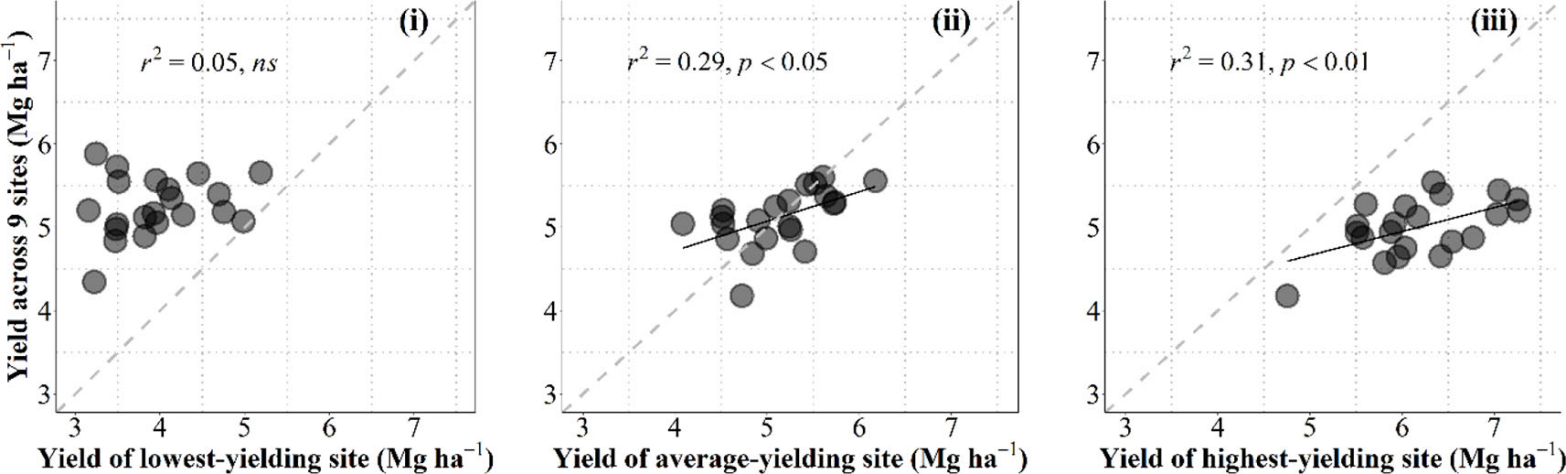
Relationship between the overall average yield across all environments but the one being used as independent variable and yield under the lowestyielding, mean- and highest-yielding conditions across the study (from leaf to right) for the 21 cultivars grown in 10 environments of Kansas produced by the combination of locations, growing seasons and management systems. The dashed line stands for the line representing Y = X (i.e., the 1:1 ratio) and solid lines represent the linear regression (when significant).

Data collected in the current study allowed us to discuss on the convenience for breeding programs to select in growing conditions that are as close as possible to those of the target environments in which released cultivars are to be grown or otherwise under better growing conditions (i.e., within the best yielding conditions that can be expected in the region). For this purpose we related the overall performance in the region for each individual cultivar with the yield of each cultivar in one particular condition. To take into account the overall performance of each genotype we calculated their average yield across nine growing conditions (all locations × years × management systems, but the particular condition that was used to predict the overall performance). These particular conditions were (i) the lowest-yielding environment, in which the most resilient genotypes would perform best; (ii) the growing condition producing an average yield closest to the overall average yield of the 10 environments; or (iii) the highest-yielding environment, in which the cultivars with the highest achievable yield would perform best (**Figure B1**, from left to right, respectively).

Naturally data-points fell above and below the line representing the 1-to-1 ratio in the left and right panels, respectively; and around that line when the environment used to predict the overall performance across all other environments was the growing condition with an average yield closest to the overall average yield (**Figure B1**).

Overall performance in the region was totally unrelated to the yield in the lowest-yielding condition (**Figure B1**, left panel). This implies that the specific characteristics making cultivars particularly adapted (or unadapted) to the most stressful condition did not contribute to the overall performance across the region (in fact the cultivars with the overall highest and lowest yielding were both rather low-yielding in this particular low-yielding condition (**Figure B1** left panel). Prediction of the overall performance from a single condition improved considerably (and became statistically significant) when using yield of an environment closest to the average-yielding growing condition as independent variable (**Figure B1**, middle panel). However, prediction of the overall performance from yield of the cultivars in the highest-yielding condition was even better than that from the average-yielding condition (**Figure B1**, right panel). Although each of the other environments were more stressful (with different levels of severity), it seemed that some attributes conferring water-limited yield potential somehow also produced a constitutive improved performance under lower-yielding environments.

This result justifies that breeding programs select promising lines under field conditions that are frequently higher-yielding than those targeted population of environments in which released cultivars are to be grown. This is in agreement with previous evidence advocating that the selection would be best if performed in high-yielding environments (Cooper et al., 1997). Using an environmental yielding condition representing higher than average yield of those targeted population of environments would likely increase the predictive performance (cf. middle and right panels in **Figure B1**).

This result also concurs with the idea that an improved yield potential (that can only be selected for in high-yielding conditions) would bring about improved performance under a range of environments with different degrees of stressful conditions; even though they would be less stable (as high yield potential implies strong responsiveness to better growing conditions; Calderini and Slafer, 1999) they might also perform better than lower-yield potential cultivars (Richards, 2000; Araus et al., 2008; Cattivelli et al., 2008; Ferrante et al., 2017). Indeed, wheats selected in CIMMYT for their high yield potential were released in drought environments (van Ginkel et al., 1998). Furthermore, selecting in higher-yielding conditions would also improve the efficacy of the program through increasing the achieved genetic gains. This is because the expected differences in performance are in line with the average yield of the environment and therefore increase the confidence in the selection process (van Ginkel et al., 1998) and explains why selection for yield in low-yielding conditions slows the progress achieved by the program (Blum, 1988; Richards et al., 2002). An empirical quantitative evidence of this is the reported positive relationship between the genetic gains achieved and the environmental average yield (Calderini et al., 1999; Sadras et al., 2016).

Box 2 **Difficulties for achieving significant genetic gains in yield.**

We analyzed the performance of commercial cultivars. That means that in a traditional historic analysis of yield gains (i.e., considering several decades of breeding), all of them would be uniformly grouped as “modern cultivars” which is relevant when comparing the breeding effect over long periods. However, analyzing the performance of cultivars released over a much shorter period may be relevant to determine the needs for maintaining/changing breeding strategies. Although far less common, analyses of short-term breeding effects (Chairi et al., 2018) are also done for this reason. Cultivars of the current study were released in the US southern Great Plains from 2007 to 2016.

**Figure B2:**
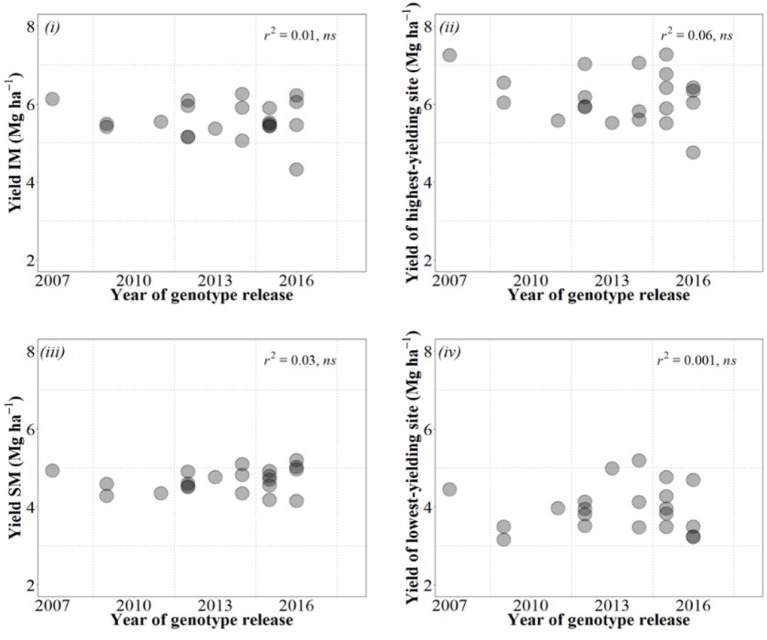
Relationships between yield of the cultivars and their year of release considering yield under IM (top left panel), SM (bottom left panel), averaged across site-years for each management system, as well as under the highest- (top right panel) and lowest-yielding condition (bottom right panel) out of the 20 combinations of site × years × management systems.

Although a decade may be a rather short period to confidently analyze the performance of breeding programs, it was worrying to see no gains in yield over the whole decade, regardless of the condition in which we estimated these gains (**Figure B2**).

This evidence that recent breeding in the US southern Great Plains failed to consistently increase wheat yield is actually further supported by a previous independent study carried out in Kansas recently in which it was shown that there were virtually no yield gains since 1992 (Maeoka, 2019). Furthermore, this does not seem to be a particular case for Kansas. Conclusions derived from some studies considering in particular the most recent yield gains from long-term breeding gains (e.g., Acreche et al., 2008; Flohr et al., 2018; Lo Valvo et al., 2018; M. Sanchez-Garcia et al., 2012) or from studies exclusively focused in the recent past (e.g., Chairi et al., 2018) indicate that recent gains in yield have been much lower than in previous decades and in some cases rather marginal or inexistent. Although part of the failure in actually increasing yields could be attributed to the fact that genetic gains in environments like Kansas, characterized by low and variable yields, are more difficult to achieve (see discussion in **Box 1**), this may not be the unique cause. The studies analyzing long periods of breeding in other low and variable yield environments (Acreche et al., 2008; Sanchez-Garcia et al., 2012; Flohr et al., 2018; Lo Valvo et al., 2018) all showed clear gains in yield from mid to late 20^th^ century, and the environments then were at least as low-yielding and as variable as they are nowadays (and for that reason they normally exhibited lower genetic gains than in high-yielding environments, but gains were clear; Calderini et al., 1999; Sadras et al., 2016). Thus, the lack of current genetic gains may well mean that a change of strategy may be required to recover the genetic gains, which are clearly needed. Identifying germ plasm possessing physiological traits that may contribute to improve yield would be ideal for strategic crosses with increased likelihood of delivering the necessary transgressive segregation required to improve yield. Thus, a physiological approach, where the physiological attributes limiting yield are recognized, complementing empirical breeding might enhance the expected gains in yield (e.g., Richards et al., 2002; Slafer, 2003).

A major conclusion from this study is that the standard management of rainfed wheat in dryland Kansas consistently fall short of achievable yields, should the management be more intensive through increasing the levels of fertilization and protecting the crop against fungal diseases. In general, yield improvement due to IM was related to a greater N uptake by the crop that brought about increases in biomass accumulation with no major changes in partitioning (and in grains per m^2^ with no compensation in average grain weight) determining a simultaneous increase in yield and protein concentration consistently across sites-years analysed. Identifying crop physiological mechanisms associated with the ability of genotypes to respond to management across different environmental conditions will help to develop efficient production systems, and assist breeding programs on the selection of genotypes with high yield potential and resource use efficiency. Hence, additional N fertilization and foliar fungicide application can help wheat producers to attain achievable yields in dryland systems *via* improving aboveground biomass and N uptake at maturity while maintaining HI.

## Data Availability Statement

The datasets generated for this study are available on request to the corresponding author.

## Author Contributions

RL and AF conceived, designed, and carried out the experiments. AS performed all data collection and analysis and drafted the manuscript. GS made substantial contributions to data analysis and interpretation, as well as manuscript writing. All authors reviewed and edited the manuscript.

## Funding

Partial funding for this research was provided by the Kansas Wheat Commission and the Kansas Agricultural Experiment Station. This research is contribution no. 20-027-J of the Kansas Agricultural Experiment Station.

## Conflict of Interest

The authors declare that the research was conducted in the absence of any commercial or financial relationships that could be construed as a potential conflict of interest.
