# Supplementary material for: Physiological Basis of Genotypic Response to Management in Dryland Wheat
Source: Front Plant Sci. 2020 Jan 10;10:1644. doi: 10.3389/fpls.2019.01644 (PMC6967739; doi:10.3389/fpls.2019.01644)
Supplement: Supplementary file 1 [file DataSheet_1.docx]

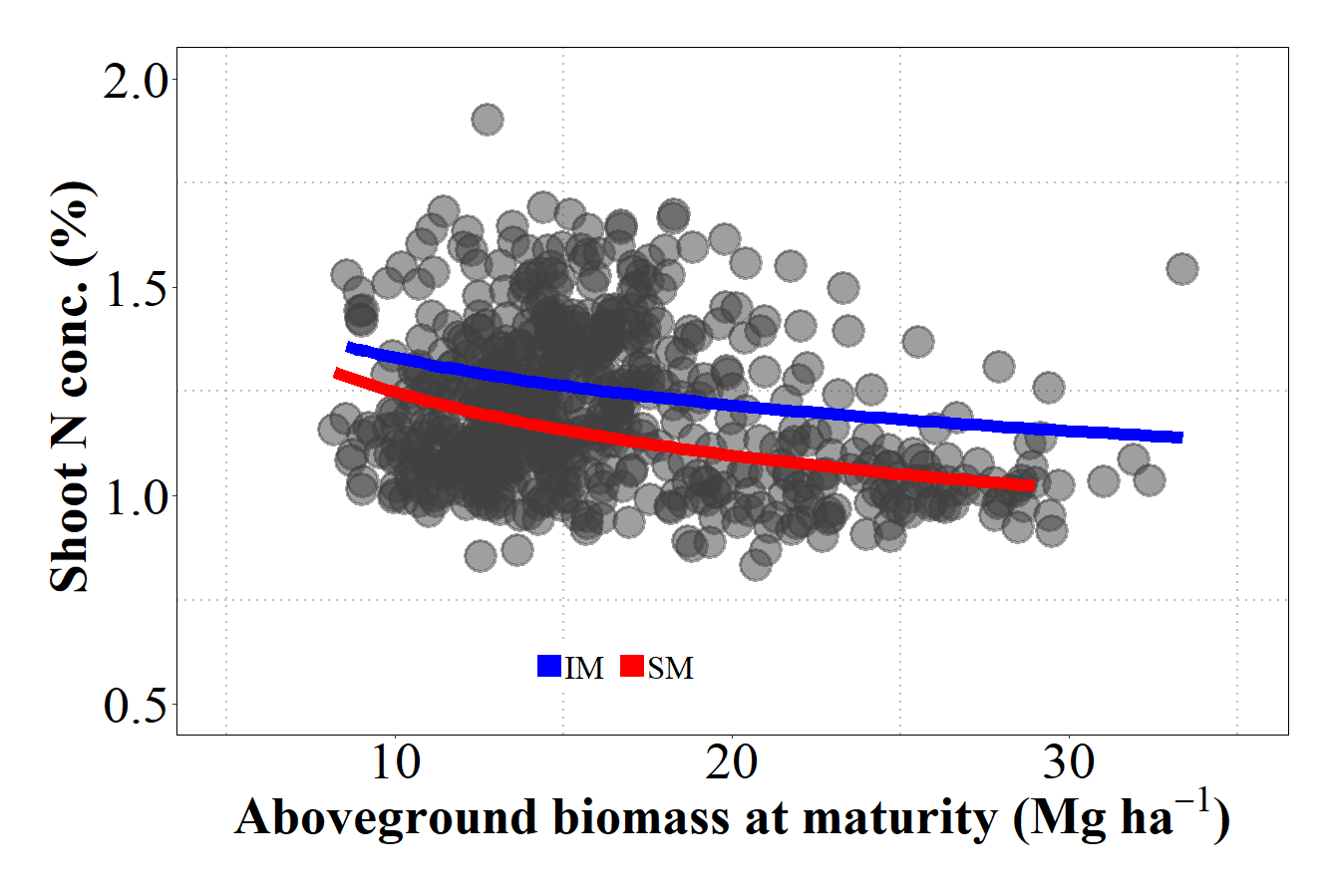


Fig S1. Shoot nitrogen concentration versus aboveground biomass at maturity across environments, genotypes and management systems intensive management (IM) and standard management (SM) (*n*=210). Power fit equation for IM Shoot N conc. = 1.79*biomass^^-0.13^. Power fit equation for SM Shoot N conc. = 1.92*biomass^^-0.18^.


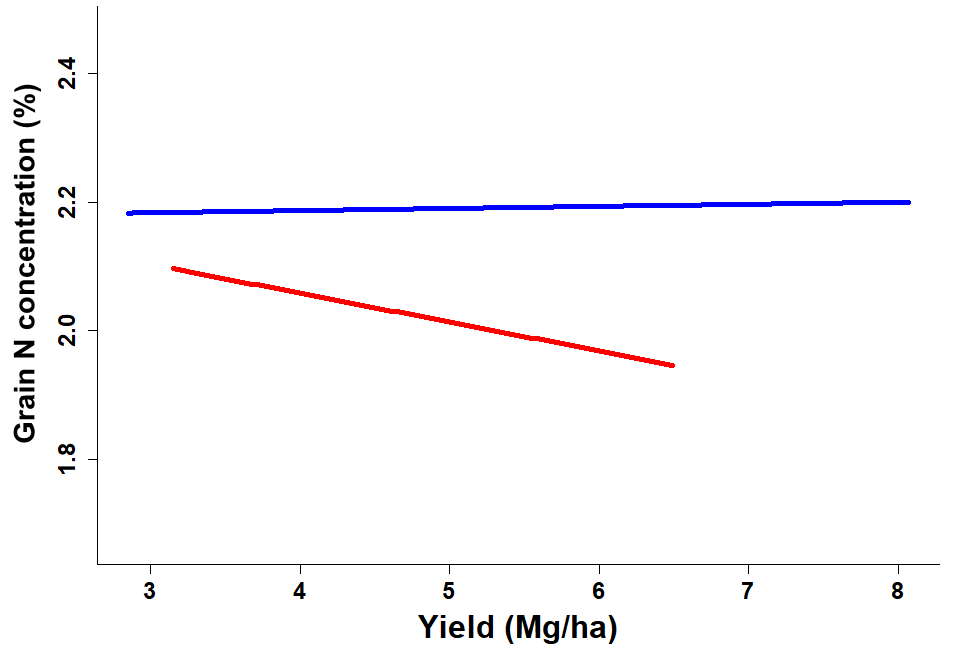


Fig S2. Grain nitrogen (N) concentration versus aboveground biomass at maturity across environments and genotypes for intensive (IM, blue) and standard (SM, red) management systems.


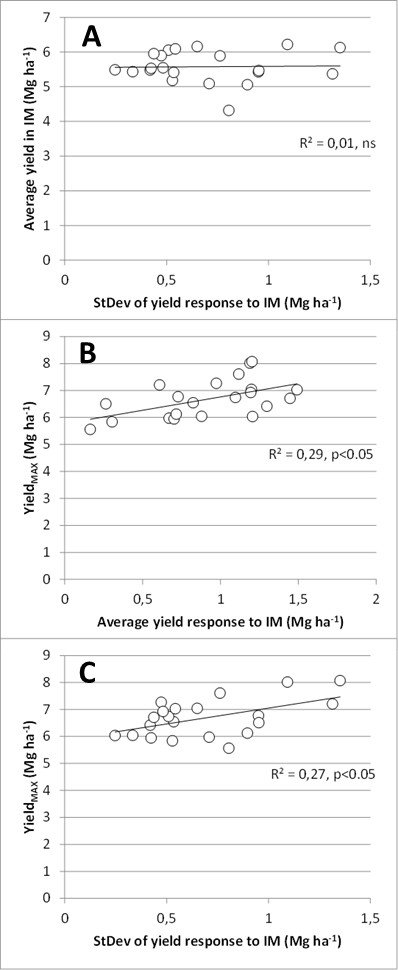


Figure S3. Relationship between mean yield in intensive management (IM) and standard deviation of yield response to IM for each of the 21 genotypes tested across site-years (A). Relationship of maximum yield values of each genotype and mean yield response to IM from all site-years (B). Relationship of maximum yield values of each genotype and standard deviation of yield response to IM from all site-years (C).

Table S1. Summary of standardize major axis (SMA) regression analysis for the relationship between grain nitrogen concentration and aboveground biomass at maturity. Differences in slopes and intercepts among management were individually analyzed. **^a^** Slope differences were estimated by likelihood ratio statistical test (Ho: slopes are not equal) forcing intercept to the origin at *p<0.05* level. **^b^** Differences in intercept among management were analyzed by the Wald statistical test (assuming same slopes) at *p<0.05* level.

| **Management** | **R^2^** | **slope (95%CI)** | **^a^statistic for slope (95%CI)** | **Intercept (95%CI)** | **^b^statistic for intercept (95%CI)** |
| --- | --- | --- | --- | --- | --- |
| IM | 0.0004 | 0.003 (-0.03; 0.04) | 0.38 (0.37; 0.40) | 2.17 (1.98; 2.36) | 2.26 (2.10; 2.42) |
| SM | 0.03 | -0.04 (-0.09; 0.002) | 0.43 (0.41; 0.44) | 2.24 (2.01; 2.46) | 2.08 (1.96; 2.22) |
